# The effects of habitual functional training on physical functioning in patients after hip fracture: the protocol of the HIPFRAC study

**DOI:** 10.1186/s12877-016-0398-8

**Published:** 2017-01-17

**Authors:** Kristi Elisabeth Heiberg, Vigdis Bruun-Olsen, Astrid Bergland

**Affiliations:** 1 Clinic of Bærum Hospital, Department of Medical Research, Bærum Hospital, Vestre Viken Hospital Trust, 3004 Drammen, Norway; 2 Oslo and Akershus University College, Oslo, Norway

**Keywords:** Exercise, Frailty, Hip fracture, Rehabilitation

## Abstract

**Background:**

The survivors after hip fracture often report severe pain and loss of physical functioning. The poor outcomes cause negative impact on the person’s physical functioning and quality of life and put a financial burden on society. Rehabilitation is important to improve physical functioning after hip fracture. To maintain the continuity in rehabilitation we have an assumption that it is of utmost importance to continue and progress the functional training that already started at the hospital, while the patients are transferred to short-term stays in a nursing home before they are returning to home. The aim presently is to examine the effects of a functional training program, initiated by the physiotherapist and performed by the nurses, on physical functioning while the patients are at short term stays in primary health care.

**Methods/design:**

Inclusion and randomization will take place during hospital stay. All patients 65 years or above who have sustained a hip fracture are eligible, except if they have a score on Mini Mental State (MMS-E) of less than 15, could walk less than 10 m prior to the fracture, or are terminally ill. The intervention consists of additional functional training as part of the habitual daily routine during short term stays at nursing homes after discharge from hospital. The primary outcome is physical functioning measured by the Short Physical Performance Battery (SPPB). Secondary outcomes are Timed “Up & Go” (TUG), hand grip strength, activPAL accelerometer, and self-reported measures like new Mobility Score (NMS), Walking Habits, University of California Los Angeles (UCLA) activity scale, Fall efficacy scale (FES), EuroQol health status measure (EQ-5D-5 L), and pain.

**Discussion:**

Issues related to internal and external validity in the study are discussed. The outline for the arguments in this protocol is organized according to the guidelines of the Medical Research Council (MRC) guidance on how to develop and evaluate complex interventions.

**Trial registration:**

ClinicalTrials.gov NCT02780076.

## Background

Hip fracture (a fracture of the upper part of the femur), is a worldwide health problem in old age [1, 2]. World-wide, there are approximately 1.7 million hip fractures each year. The highest rates are seen in North America and Europe [1]. In Norway (with 4.7 million inhabitants), about 9000 patients are hospitalized and operated for hip fractures annually. This is among the highest reported hip fracture incidence rates in the world [1–3], even though the hip fracture rates in Norway have declined during the past 15 years. Nevertheless, the forecasted growing number of older people might cause an increase in the absolute number of fractures. This may lead to a substantial, societal, economic and public health burden [4]. Hip fracture is a serious event; one out of every four patients die within one year. Survivors often report severe pain and loss of physical functioning [3].

In a systematic review it was estimated that 42% of survivors after hip fracture did not return to their pre-fracture level of physical functioning, and 35% became incapable of walking independently after the fracture [5]. Moreover, 50% of those able to walk without a walking device before the fracture became permanently dependent on a walking device, and 50% of survivors had persistent loss of independence in daily activities after the fracture [6, 7]. Long-lasting mobility limitations after hip fracture may lead to prolonged physical disability, increased fall risk and new injuries, and 15% of the patients will become permanently institutionalized [7]. Altogether, these impairments cause considerable suffering. Therefore, strategies for hip fracture rehabilitation to enhance the patients’ recovery of physical functioning and prevent future falls is an urgent health care challenge. With such serious consequences, prevention and treatment of fractures to improve outcomes are crucial.

In Norway, the mean length of hospital stay (LOS) after hip fracture surgery is 5–6 days [8]. An increasingly shorter LOS is in line with international studies showing that aging patients are discharged from the hospital “quicker and sicker” [9, 10]. After discharge, most of the patients in Norway are transferred to short-term stays in nursing homes in primary health care before they are able to return to home.

Functional training, such as walking and transfers, ought to be an important part of the rehabilitation after hip fracture. Nevertheless, research indicates that rehabilitation strategies after hip fracture vary [11–13]. Experts agree that for better survival rate and for improvement of pain, quality of life and physical functioning, early assisted ambulation should begin while the patients are in hospital [10–12]. According to a systematic review and two Cochrane reviews [14–16], there are no set guidelines for best practice training programs after discharge from hospital. To maintain the continuity in rehabilitation, we have an assumption that it is of utmost importance to continue and progress the functional training during the sub-acute phase while the patients are at short-term stays in nursing homes. However, in the nursing homes the resources of physiotherapists and nursing staff seem to be limited, and the nursing staff involved may only to a small extent be focused upon enhancement of the patients’ physical functioning. These assumptions are supported by a study that examined the nurses’ experiences and attitudes towards rehabilitation after hip fractures [17]. The nurses working in the nursing homes, as opposed to the nurses at the hospital, seemed to be less concerned with their role and participation in the patients’ rehabilitation process [17]. Therefore, the possible discontinuity in the rehabilitation efforts during short-term stays may have a negative impact on the patients’ recovery of physical functioning.

Progressive resistance training is found effective in improving physical functioning in the frail elderly [18] and in patients after hip fracture surgery [13, 19]. However, progressive resistance training may be difficult to carry out in the sub-acute phase, partly because of limited recourses, and partly because of the patients’ pain and health condition after surgery. An option may therefore be to continue and progress the functional training started during hospital stay, such as training in walking and further on repetitive sit-to-stands, as part of the daily habitual routine during their short-term stays in the nursing homes [20]. This type of functional training may be motivational and easily recognizable to the patients, and it can also be carried out by the nursing staff with only initial guiding from a physiotherapist. There seems to be lack of knowledge on the effect of additional functional training, incorporated as part of the habitual daily routine during short-term stays, on the patients’ immediate and long term recovery of physical functioning and activity level after hip fracture, compared to usual care alone.

### Aims

#### Primary aim

- To assess the immediate and long-term effects of an additional functional training program, performed in a population of frail older patients with low energy hip fracture, as part of the habitual daily routine during their short-term stays in nursing homes, on physical functioning measured by the Short Physical Performance Battery (SPPB).

#### Secondary aims

- To assess the immediate and long-term effects of the same program on ambulation, level of physical activity, health-related quality of life, pain in rest and while walking, fear of falling, number of secondary falls, support from health care services, place of residence, mortality rate, and the cost-effectiveness of the intervention.

## Methods/Design

### Project context

The present study is undertaken at Bærum Hospital Vestre Viken and in Bærum municipality, located nearby Oslo, Norway. Bærum Hospital serves as a local hospital for about 300 000 inhabitants, and approximately 350 patients are treated surgically for hip fractures at the hospital each year. The patients are hospitalized in the orthogeriatric section within the Department of Orthopedic Surgery. An orthogeriatric approach involves several medical- and healthcare disciplines, such as geriatrician, orthopedic surgeon, nurse, physiotherapist, occupational therapist, and pharmacist [21–23]. The team examines the patients and evaluates their medical-, functional-, and cognitive status, risk of secondary falls, and need for further rehabilitation. An important part of the treatment during hospital stay is early mobilization, such as getting out of bed and walk within 24 h after surgery. The patients receive physiotherapy exercises each day from Monday to Friday. Additionally, the nurses mobilize the patients on each shift during all week days.

Due to the short hospital LOS, most of the patients are in need of short-term stays in nursing homes for 2–3 weeks, before they are able to return to home. During the short-term stays the patients usually get in contact with a physiotherapist 2–3 times a week. Physiotherapy includes mobilization and gait training [24]. The intervention presented in this protocol contains additional functional training initiated by the nurses and performed at least 4 times a day as part of the daily routine.

### Study design

The study is designed as a single-blind randomized controlled trial (RCT), comparing the effects of additional functional training to usual care alone during short-term stays in nursing homes. This design is the gold standard to test effects of interventions [25, 26].

### Recruitment and study population

Recruitment takes place at Bærum Hospital after hip fracture surgery. Patients with an acute low-energy hip fracture (intracapsular, throchanteric or subtrochanteric) and treated surgically, ≥ 65 years of age, living in their own homes prior to the fracture, and able to give an informed consent, are invited to participate in the study. Patients are excluded if they are unable to walk 10 m with or without a walking aid prior to the fracture, have a score of less than 15 points on Minimal Mental Status Evaluation (MMS-E) in the acute phase, have a pathological fracture, life expectancies of less than 3 months, medical contraindications for training, or are incapable of understanding and speaking Norwegian.

### Randomization and allocation concealment

Four nursing home departments in Bærum municipality that offer short-term stays after hip fracture are included in the study. First, the nursing home departments are matched into two similar groups in terms of anticipated number of patients and culture. Thereafter, the two groups are randomly allocated to either an experimental group that contains a functional exercise program in addition to usual care or to usual care alone (control). At discharge from hospital, the participants are consecutively allocated to the nursing home with the first vacancy. The participants are not in a position to have any influence on the allocation.

### The interventions

#### The functional training group (experimental group)

The patients who are allocated to the experimental group are receiving a functional training program initiated by the nurses in addition to the usual physiotherapy. The present functional training program is based on transfer activities of daily life, tailored to meet each individual’s needs. It is targeted to the level of difficulty relevant for each participant. The functional training program is performed approximately 4 times a day, 7 days a week during the short-term stay of 2–3 weeks. The nurses will be educated, motivated, guided, and frequently supervised by the research physiotherapists.

The functional training program is inspired by prior research on the effect of a walking skill training program based on ambulatory activities and transfers in patients with total knee [27] and total hip [28] arthroplasty. These patients changed statistically significantly more in walking capacity measured by the 6-min’ walk test, compared to controls. The program is also inspired by the gait and balance program developed by Thingstad et al [29] and performed in patients 4 months after the hip fracture.

The content of the present functional training program is as follows:

- *Stepping.* Standing with support, weight transfer without stepping. Progression: Short steps forward and sideways, less support, several repetitions in series of 3.
- *Walking in the corridor with a walking aid.* Walk several short distances. Rest on a chair if necessary. Progression: Walk with longer steps, good stride, higher speed, start, stop, and turns. Walk in the corridor as long as you are able to.
- *Step-up.* Tapping on to the step but not stepping up. With support: stepping up one or several steps. Progression: Climb up and down the staircase.
- *Chair rise.* Raising/lowering on heightened chair with arm support. Progression: Raising/lowering on normal chair with less or without arm support, several repetitions in series of 3.
- *Squats.* Stand with arm support. Bend your knees by lowering the body. Progression: Less arm support, deeper bends, several repetitions in series of 3.
- *Heel lift.* Stand with arm support. Several repetitions in series of 3.

When transferred to home, the patients are encouraged to continue their exercises at minimum twice daily. They are given a written exercise program with progression before discharge from the nursing homes.

#### The control group

The patients who are allocated to the control group are receiving treatment as usual; physiotherapy 2–3 times a week during their short-term stays.

### Measures

Assessments will be performed before discharge from hospital (baseline), when the patients have stayed at the nursing home for 2 weeks, and at 3 and 12 months after surgery (Table 1). The 3- and 12-month assessments will be performed at Bærum Hospital or in the patient’s home if necessary. Blinded assessors (experienced physiotherapists) will perform the assessments. Before the study is initiated, the testers are taking part in a training program regarding testing procedures in order to ensure high inter-rater test reliability.

**Table 1:** Data collection at different time points in the HIPFRAC study

	4-5 days after surgery	3 weeks after surgery	3 months after surgery	1 year after surgery
*Personal variables*				
Age	x			
Sex	x			
Education	x			
Cohabiting status	x			
Comorbidity	x			
Type of operation	x			
History of falls	x			
Minimal mental status evaluation (MMS-E)	x			
*Performance-based measures*				
Short Physical Performance Scale (SPPB)	x	x	x	x
ActivePAL		x		
Timed Up & Go (TUG)	x	x	x	x
Grip strength	x			x
*Self-reported measures*				
New mobility score (NMS)	x (recalled from prefracture)			^X^
Walking habits	x (recalled from prefracture)		x	x
Health-related quality of life (EQ-5D-5 L)	x	x	x	x
Pain at rest and while walking	x	x	x	x
University of California Los Angeles (UCLA) activity scale	x	x	x	x
Fall efficacy scale (FES)	x	x	x	x

#### Primary outcome of physical functioning

*Short physical performance battery (SPPB)* is a measure of physical functioning [30]. SPPB evaluates balance, mobility and muscle strength by examining an individual’s ability to stand in different positions, time to walk 4 m, and time to rise up from and sit down on a chair 5 times [30]. The tests are scored between 0 and 4, leaving a maximum score of 12 [30]. The SPPB will be applied before discharge from hospital, after 14 days in the nursing home, and 3 and 12 months after surgery.

### Secondary outcomes

#### Performance -based measures of physical functioning

*Timed Up &Go (TUG)* is a measure of mobility. The patient gets up from a chair with armrest, walks forward for three meters, turns, walks back and sits down in the chair [31, 32]. The time is recorded in seconds and measured before discharge from hospital, after 14 days in the nursing home, and at 3 and 12 months after surgery.

In the *stair climbing test* the patients ascend and descend eight steps with a step height of 16 cm with alternate legs while holding onto the rail. The time is recorded in seconds and the test will only be recorded at 3 and 12 months.

*Single-axis accelerometers (activPAL)* assess the patients’ physical behavior during short-term stays in the nursing homes. The accelerometer is attached to the patients’ non-affected thigh before discharge from hospital. Mean upright time and mean upright events during the first week in the nursing homes are measured. The nurses remove the devices after 14 days.

*A hand dynamometer* is used to measure grip strength before discharge from hospital and at 12 months. The grip strength is associated with general muscle strength and walking recovery, and it might provide important prognostic information regarding the patients’ future functional trajectory [33].

#### Self- reported measures of physical functioning, physical activity and quality of life

*New mobility scale (NMS)* measures pre-fracture physical functioning. The NMS classifies the pre-fracture physical functioning as either low (from 2–6) or high (from 7–9) [34]. It is used only in the acute phase while the patients are in hospital.

*Walking Habits questionnaire* measures the frequency and duration of the patient’s weekly walking habits pre-fracture and at three and 12 months after surgery [35, 36].

*Fall Efficacy Scale (FES)* measures the patient’s fear of falling [37, 38]. Sixteen questions are scored on a 4-point scale ranging from “not at all concerned” to “very concerned” about falling on pre-fracture level and at 3 and 12 months.

*The University of California, Los Angeles (UCLA) Activity Scale* measures the patients’ level of physical activity [39]. The patient’s physical activity is evaluated on a 10-point scale based on 10 descriptive activity levels ranging from wholly inactive and dependent to regular participation in impact sports. Pre-fracture-, 3- and 12-month activity level will be measured.

*Health and Quality of Life Questionnaire (EQ-5D-5 L)* measures the patient’s health-related quality of life [40, 41]. Five questions are scored on a 5-point scale. Additionally, the self-rated health is reported on a vertical, visual analogue scale. Cost-qualy analysis will be provided from EQ-5D-5 L. The measures will be taken before discharge from hospital, after 14 days in the nursing home, and at 3 and 12 months after surgery.

#### Sociodemographic - and other variables

Age, sex, living arrangements, educational level, previous falls, home care services, medication list, date of hip fracture, and type of operation are variables that are collected at baseline. Information about number of falls, readmission to hospital, and mortality are also registered at discharge from the nursing homes, at 3 and 12 months after surgery.

### Adherence to the program

To ensure adherence to the program, a physiotherapist employed in the project will educate and motivate the nursing staff in the nursing homes in the functional training group. The nurses will register in a log when they have performed the functional training, at least 4 times a day. Additionally, the accelerometer registration will reveal the activity level in both groups.

### Adverse advents

Adverse events are registered in the following 4 categories: falls, cardiovascular events, musculoskeletal injuries and health care utilization [42]. Furthermore, number of scheduled training sessions and reasons for non-attendance are registered. Readmission to hospital will also be recorded.

### Data analysis and statistical power

The sample size is calculated from a small meaningful change in SPPB, the primary outcome measure [43]. A small meaningful change in the SPPB is mean (SD) 0.5 (1.48) points [44]. With a significance level of 0.05 and power of 80% this estimate requires 140 patients, 70 patients in each group. To compensate for potential drop-outs at least 160 patients will be included in the study. All data will be stored at the research server at the hospital. Between-group differences in normal distributed continuous variables will be analyzed with *t*-tests and/or analysis of covariance (ANCOVA) according to the principle of intention-to-treat (ITT). Missing data will be imputed by carrying the last value forward. Effect sizes will be calculated. To examine relationships correlation and/or multiple regression analyses will be used [45]. The significant level is set to *p* < 0.05.

### Time plan of the study

Recruitment will start during spring 2016 and will probably be finished by the end of 2017. The intervention will be ongoing in the same time interval. Data collection will last for 1 year after recruitment. Thereafter, we will write up and publish peer-reviewed articles.

### Ethics and approvals

Before discharge from hospital, an informed consent will be obtained if the physiotherapist who is recruiting is reassured that the patient fully understands what it implies to participate. The patients with increased risk of falls, restrictions in movement or weight-bearing during training and activity will be focused upon to avoid injuries. The nursing staff will be trained to secure the patients properly to avoid falls and dislocations. All patients recruited to the project will also be offered usual care rehabilitation. The study is funded from Vestre Viken Hospital Trust and is approved by the Regional Committee for Ethics in Medical Research (South-East Norway) (2015/2147). ClinicalTrial.cov NCT02780076.

## Discussion

We expect that the intervention described in this protocol will have positive impact on the physical functioning measured by SPPB. The tailored intervention will have potential to promote evidence-based decision-making and empower patients with hip fracture to remain in charge of their own lives. We rely on a systematic approach which corresponds with the guidance on developing and evaluating RCTs [25, 26].

This protocol creates a plan for the investigators to follow. Several issues related to the quality of the study have to be discussed, such as internal and external validity. Internal and external validity relates to whether the results drawn from the study can be trusted and generalized into other contexts. Examples of methodological issues that may influence internal validity in a pragmatic RCT include random allocation, blinding, outcome measures, sample size, drop-outs, statistical methods, and the participants’ adherence to the intervention.

In the present study we are particularly concerned with the outcome measures and the participants’ adherence to the program. A standard set for the measurement of outcomes in patients with hip fracture has been identified [46]. In line with their recommendations we chose to focus on mobility, level of activity, and health-related quality of life measures, and also include some other outcomes, such as pain, fear of falling, number of falls, complications and mortality rate. The intervention is designed to increase the amount of physical activity, which in turn may increase the patients’ physical functioning, such as their ability to move around. Therefore, the primary outcome is physical functioning measured by the SPPB. The SPPB has also been used in prior research to measure effects of interventions on physical functioning. In one study the SPPB was used to examine the effect of comprehensive geriatric care, compared to orthopedic care, in the acute postoperative phase during hospital stay [22]. The SPPB was responsive and able to reveal a difference between the groups even a few days after surgery. Our primary outcome measure is therefore considered to be sensitive to change, responsive and also used in other comparable studies. Our results may therefore be comparable to others, and this will strengthen the validity of the study.

Other outcome measures are also important to apply to give a broad picture of the patients’ health and life situation after hip fracture. The self-reported measurements of activity level and fear of falling will add information on how the patients themselves consider their present activity level and concerns related to future falls. Registration of home care services will give a picture of their ability to manage daily activities. Furthermore, in EQ-5D-5 L the patients’ health-related quality of life, including depression and anxiety, will reveal other important aspects of the recovery of physical functioning after hip fracture. In the present study both performance-based and self-reported outcome measures are used. The different measurements will strengthen the study as they are complementing each other in terms of measuring the functional status in a broad sense [47]. To maximize the test-retest reliability, the assessors are experienced physiotherapists who are properly trained in testing before the inclusion starts.

Gait and balance are important aspects of mobility in everyday life. Moreover, research has shown that functional exercises, which involve shifts between positions with weight-bearing over a changing base of support, are important to improve gait and balance in older people and thereby reduce the fall risk [48, 49]. There is a close association between mortality rate and level of physical functioning, such as mobility [50], which underlines the importance of increased physical activity for the patients with hip fracture. Prior studies have focused on the effect of functional-, strength- and balance exercises several months after hip fracture [13, 16, 29]. To our knowledge, little research has been done on the effect of easily adoptable functional exercises while the patients are at short-term stays in a nursing home after hip fracture. This is a period when the patients have just started their recovery process, but are still vulnerable and in need of support to improve further. In general, exercises are believed to depend on certain intensity with respect to repetitions and exercise duration time to exert their effects. In the meta-analysis of Diong et al [13] they found that the interventions with the best effects were delivered later in the recovery course and with longer duration than the present one. Therefore, it will be interesting to examine whether our functional training intervention, performed 4 times daily for 2 weeks in the early sub-acute phase, is of sufficient length and intensity to exert an effect.

To further take care of the internal validity of the study, an apparent challenge is the adherence to the program. That is whether the patients are capable of adhering to the program and whether the nurses will adhere and keep on initiating the program despite their already heavy workload. We assume that the nurses may get a busier working schedule during the first days of the patients’ short-term stays, but thereafter their workload may be reduced, as the patients get stronger, more mobile and possibly will manage to do functional activities and the functional training by themselves. Even so, adherence to the program is of utmost importance and a challenge that will require motivational efforts from all persons involved.

External validity or generalizability is another important issue to discuss. In this study we include patients who are living at home before the fracture, but are in need of short-term stays after discharge from hospital before they are able to return to home. Those returning directly to home after discharge from hospital are excluded. Also those who are physically or mentally too impaired to manage the training intervention and completing the measurements are excluded from participating. Therefore, the results can only be generalized to a similar group and neither to the healthiest who return directly to home, nor to those that were institutionalized before the fracture.

The present experimental intervention is considered to be a complex intervention. A complex intervention in health care is defined according to the number of interacting components involved [51]. The outline for the arguments in this protocol is organized according to the guidelines of the Medical Research Council (MRC) guidance on how to develop and evaluate complex interventions [51]. According to the new Medical Research Council guidance, certain issues have to be taken into account when reporting such interventions. The different components within the interventions, how the intervention is tailored to the local context, and how the intervention is implemented are all important factors to describe in detail. A description of these elements may broaden the understanding of possible other active components within the intervention and thereby add knowledge to factors that may have contributed to the eventual effect [26]. These issues are taken into consideration and elaborated on when reporting on the effects from the randomized controlled trial, but are not included in this protocol.

Running intervention studies for multi-diseased fracture patients with low functional capacity is challenging with regards to data collection. Nevertheless, it is important to run such studies to be able to develop interventions that may improve wellbeing and minimise functional decline in these patients. Prior studies have often used small samples and a pragmatic choice of interventions [13]. The present study has, due to its large sample size and theoretically based intervention, the potential to generate new knowledge that may improve the design of future activity programs for patients with hip fracture. It is our hope that knowledge drawn from this study may contribute to fill the gap between hospital, primary health care and home in the rehabilitation of the frail elderly after hip fracture.

## Abbreviations

activePAL: Single-axis accelerometer
ANCOVA: Analysis of covariance
EQ-5D-5 L: Health and quality of life questionnaire
FES: Fall efficacy scale
ITT: Intention to treat
LOS: Length of stay
MMS-E: Minimal mental status evaluation
MRC: Medical research council
NMS: New mobility scale
RCT: Randomized controlled trial
SD: Standard deviation
SPPB: Short physical performance battery
TUG: Timed up & go
UCLA: University of California, Los Angeles
